# Salicylic Acid-Induced Elicitation of Nepetalactone and Rosmarinic Acid Biosynthesis in Naked Catmint (*Nepeta nuda* L.): Metabolomic and Transcriptional Insights

**DOI:** 10.3390/ijms27083570

**Published:** 2026-04-16

**Authors:** Luka Petrović, Slavica Dmitrović, Jasmina Nestorović Živković, Biljana Filipović, Neda Popović, Milica Milutinović, Dragana Matekalo, Uroš Gašić, Danijela Mišić, Marijana Skorić

**Affiliations:** Department of Plant Physiology, Institute for Biological Research “Siniša Stanković”—National Institute of the Republic of Serbia, University of Belgrade, Bulevar Despota Stefana 142, 11108 Belgrade, Serbia; slavica.dmitrovic@ibiss.bg.ac.rs (S.D.); biljana.nikolic@ibiss.bg.ac.rs (B.F.); dragana.bozic@ibiss.bg.ac.rs (D.M.); uros.gasic@ibiss.bg.ac.rs (U.G.); dmisic@ibiss.bg.ac.rs (D.M.)

**Keywords:** salicylic acid, *Nepeta nuda*, nepetalactone, rosmarinic acid

## Abstract

Salicylic acid (SA) is involved in plant defense responses to environmental stressors by modulating gene expression and specialized metabolites production, enhancing plant adaptive resilience through systemic signaling pathways. This study investigates the impact of exogenous application of SA on the metabolism of iridoids and phenolic compounds—characteristic specialized metabolites of the *Nepeta* species, associated with diverse biological activities. Nepetalactone (NL) is a characteristic monoterpene iridoid, while rosmarinic acid (RA) represents the most abundant phenolic compound within the genus. We explored the effects of varying SA concentrations (2 µM, 5 µM, 10 µM, and 20 µM) on iridoid and phenolic metabolism in in vitro-grown *Nepeta nuda*, following 7 days and 28 days of elicitation. A significant increase in *trans*,*trans*-NL content was observed after 7-day exposure to 2 µM SA, while prolonged exposure led to a decrease in its levels, particularly at higher SA concentrations. Gene expression analysis revealed that 7 days of exposure to lower concentrations of SA upregulated genes coding for NAD-dependent nepetalactol-related short-chain dehydrogenase/reductases (NEPSs), key regulatory enzymes catalyzing the final steps of NL biosynthesis. In contrast, prolonged exposure to 20 µM SA downregulated genes coding for geraniol 8-hydroxylase (NnG8H) and 8-hydroxygeraniol oxidoreductase (Nn8HGO), which resulted in reduced iridoid content. Conversely, SA treatment notably increased RA content after prolonged exposure to 20 µM SA, which is a result of the enhanced expression of all analyzed RA biosynthesis-related genes. These findings demonstrate that both concentration and duration of SA treatment are critical determinants of elicitation outcomes in *N. nuda*. Strategic manipulation of these parameters can redirect metabolic flux toward either iridoid or phenolic compounds production, and enhance biotechnological production of specialized metabolites in *N. nuda*.

## 1. Introduction

Nepetalactone (NL) is a characteristic and predominant monoterpene iridoid in the genus *Nepeta*, which has gained considerable attention for its wide-ranging biological activities. In particular, NL exhibits potent insect-repellent properties [1], making it a promising candidate for the development of eco-friendly biopesticides. Furthermore, NL has demonstrated phytotoxic effects, indicating its potential for use as a bioherbicide in sustainable agricultural practices [2,3,4]. Notably, *Nepeta* species produce multiple stereoisomers of NL and the specific quantity and ratio of these isomers determine the effectiveness as insect repellents [5]. Numerous studies have confirmed the efficiency of *Nepeta* essential oils and their bioactive NL isomers in providing both spatial and contact repellency against various mosquito species [6,7,8]. The effectiveness of these compounds presents significant potential for the development of repellent formulations for the pharmaceutical industry and pest management. This is particularly crucial in addressing the growing threat of mosquito-borne diseases in animals and humans, and in preventing the expansion of invasive insects-transmitted plant diseases, which are accelerated by climate change and human activity [9,10].

*Nepeta* leaves are characterized by the presence of various phenolic acids from the group of hydroxycinnamic acids, such as caffeic acid (CA), chlorogenic acid (CGA), and rosmarinic acid (RA), the last one being the most abundant in the majority of *Nepeta* species [11]. This is also the case for *Nepeta nuda* [12,13]. RA is well-known for its potent antioxidant properties, which are crucial in protecting cells from oxidative stress, reducing inflammation, and in promoting wound healing [14,15]. Additionally, RA has demonstrated strong antiviral and antibacterial activities [16,17,18], emphasizing the traditional use of *N. nuda* for treating infections [19].

The populations of many *Nepeta* species, particularly endemic ones, are rapidly declining due to a range of factors including habitat destruction, competition, overgrazing, erosion, land clearance, and other human activities [20]. In addition, rapid climate change poses increasing threats to natural populations. Many *Nepeta* species appear sensitive to environmental stresses and many face low biomass production, which seems to be an obstacle for its large-scale cultivation and commercialization. However, one of the most widely distributed *Nepeta* species, *N. nuda*, proved to be a highly resilient plant, often growing in ruderal habitats alongside roads, with a significant biomass production [21]. NL is commonly found among specialized metabolites of naked catmint, which is a common name of *N. nuda*, although its content in extracts and essential oils can vary significantly depending on geographical distribution and environmental factors [19,21,22].

Salicylic acid (SA) is a well-known phenolic phytohormone that plays a significant regulatory role in plant growth and development, and in plant responses to environmental stimuli. It influences numerous physiological processes such as seed germination, stomatal movements, pigment accumulation, photosynthesis, enzyme activity, nutrient uptake, flower induction, and membrane functions [23,24,25]. SA is also a key signaling molecule that enhances plant tolerance to both abiotic [26,27,28] and biotic stressors [29,30,31]. Exogenous SA application effectively modulates plant responses to various stresses, including water deficit [32,33], UV-B radiation [24], salinity stress [34], low temperature stress [35], and heat stress [36]. SA coordinates plant defense mechanisms in response to abiotic and biotic environmental stimuli by activating various signaling pathways, modifying gene expression, and promoting the biosynthesis of specialized metabolites, thereby strengthening plant resilience and adaptive plasticity [37,38]. It has also been proven that SA can have a significant impact on specialized metabolite production, including iridoids and phenolics [39,40]. The effects of SA were previously examined by evaluating the growth and chemical composition of *N. cataria*, a close relative of *N. nuda* [39,41]; however, it remained unclear how long-term exposure and varying concentration of SA affect iridoid and phenolic metabolism in *Nepeta*. Moreover, this study represents the first investigation of SA impact on iridoid and phenolic content of *N. nuda* grown under in vitro conditions, allowing controlled evaluation of SA-induced changes in iridoid and phenolic metabolism. The elucidation of the molecular mechanisms underlying metabolic responses of *N. nuda* plants to SA treatments, with particular focus on NL- and RA-related biosynthetic genes, further opened new avenues for developing strategies for enhanced metabolite production in other *Nepeta* species. Understanding the physiological and biochemical responses of plants to exogenous SA can help develop effective strategies to improve plant resilience under changing environmental conditions and may enable more effective stress management in plants, supporting sustainable agriculture and food security.

## 2. Results and Discussion

**Figure 1:**
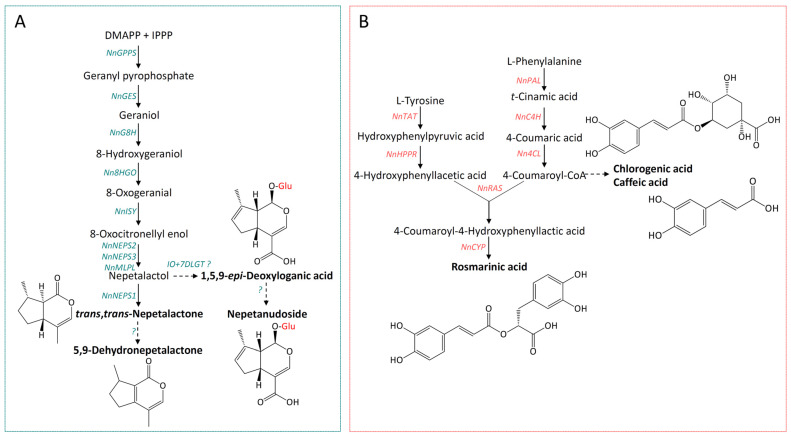
(**A**) Proposed biosynthetic pathway of major nepetalactone-type and glycosilated iridoids in *Nepeta nuda*. (**B**) Simplified scheme of the biosynthetic pathway of major phenolic acids in *Nepeta*. Compounds presented in bold letters are quantified in this study. Iridoid biosynthesis-related genes analyzed in this study are marked with blue letters, while rosmarinic acid biosynthetic genes are presented as red letters. Abbreviations: GPPS—geranyl diphosphate synthase; GES—geraniol synthase; G8H—geraniol 8-hydroxylase; 8HGO—8-hydroxygeraniol oxidoreductase; ISY—iridoid synthase; NEPS—NAD-dependent nepetalactol-related short-chain dehydrogenase/reductase; MLPL—major latex protein-like enzyme; IO—iridoid oxidase; 7DLGT—7-deoxyloganetic acid glucosyltransferase; PAL—phenylalanine ammonia-lyase; C4H—cinnamic acid 4-hydroxylase; 4CL—4-coumaric acid CoA-ligase; TAT—tyrosine aminotransferase; HPPR—hydroxyphenylpyruvate reductase; RAS—4-coumaroyl-CoA:4′-hydroxyphenyllactic acid 4-coumaroyltransferase; CYP—cytochrome P450-dependent monooxygenase.

All *Nepeta* iridoids are synthesized through the core iridoid pathway up to nepetalactol (Figure 1A). From this point, the biosynthesis of NL-type iridoids and iridoid glucosides (IGs) diverges into separate branches. The initial biosynthetic steps are driven by several key enzymes: geranyl pyrophosphate synthase (GPPS), geraniol synthase (GES), geraniol 8-hydroxylase (G8H), 8-hydroxygeraniol oxidoreductase (8HGO), iridoid synthase (ISY), NAD-dependent nepetalactol-related short-chain dehydrogenase/reductase (NEPSs), and major latex protein-like (MLPLs) enzymes. While NEPS2, NEPS3, and NEPS4 isoforms catalyze the stereoselective formation of nepetalactol, NEPS1 and NEPS5 catalyze the oxidation of nepetalactol to NL (Figure 1A). Among NEPS enzymes, NEPS1, NEPS2 and NEPS3 were previously identified in *N. nuda* transcriptome [21]. Up to date, the biosynthetic step leading from NL to 5,9-DNL is not resolved. When it comes to the biosynthetic branch leading to IGs, it is assumed that two enzymes, iridoid oxidase (IO) and 7-deoxyloganetic acid glucosyl transferase (7-DLGT), catalyze the conversion of nepetalactol to 7-deoxyloganic acid (7-DLA) [42], although some studies indicate that biosynthesis of iridoid glycosides in *Nepeta* also requires the activity of NEPSs [43].

RA, the dominant phenolic compound in *N. nuda*, is synthesized through two distinct biosynthetic routes, the phenylpropanoid and the tyrosine pathways (Figure 1B). The phenylpropanoid branch begins with L-phenylalanine, which undergoes a series of enzymatic conversions catalyzed by phenylalanine ammonia-lyase (PAL), cinnamic acid 4-hydroxylase (C4H), and 4-coumaric acid CoA-ligase (4CL), to produce 4-coumaroyl-CoA. On the other hand, transamination of L-tyrosine by tyrosine aminotransferase (TAT) leads to the formation of 4-hydroxyphenylpyruvic acid, which is then reduced by hydroxyphenylpyruvate reductase (HPPR) to produce 4-hydroxyphenyllactic acid. The enzyme rosmarinic acid synthase (RAS) then catalyzes the formation of 4-coumaroyl-4′-hydroxyphenyllactic acid (4C-pHPL) via the conjugation of 4-coumaroyl-CoA and 4-hydroxyphenyllactic acid. A specific cytochrome P450 monooxygenase (CYP) subsequently introduces hydroxyl groups onto the 4C-pHPL molecule, thus finalizing the synthesis of RA [44].

SA has been employed in a number of studies to induce the accumulation of specialized metabolites, including iridoids [38,39] and phenolics [45,46]. This effect is commonly achieved by altering the expression of corresponding biosynthetic genes [45,46] or through modulating the regulatory role of transcription factors such as NPR1, WRKY, MYB15, bHLH, and NAC [47,48,49,50,51]. Tajik et al. [46] demonstrate that upregulation of *PAL* resulted in RA accumulation in *Crocus sativus* following elicitation with SA. Li et al. [45] show that SA can induce upregulation of genes coding for TAT, PAL, and RAS, affecting the accumulation of phenolic acids in *Salvia miltiorrhiza*. Rubio-Rodríguez et al. [40] demonstrate SA-induced accumulation of iridoids in *Castilleja tenuiflora*, associated with upregulation of 1-deoxy-D-xylulose-5-phosphate synthase (*CteDXS1*) and geraniol 10-hydroxylase (*CteG10H*) genes, the latter being directly involved in iridoid biosynthesis. In *Nepeta* species, the exogenous application of SA on specialized metabolism has also been investigated. Foliar application of SA on two catnip chemotypes (*N. cataria* and *N. cataria* var. *citriodora*) enhanced essential oil accumulation and improved plant growth under water-stress conditions [41]. More recently, Allen et al. [39] explored the short-term metabolic and molecular responses of *N. cataria* to exogenous SA treatment. The study demonstrated that supplementation of a nutrient solution with 0.5 mM and 1.0 mM SA for 24 h in hydroponically grown *N. cataria* results in slight upregulation of iridoid biosynthetic genes coding for geranyl diphosphate synthase (GPPS) and NEPS1, without affecting NL levels in plants. Given these findings, we pursued a prolonged exposure of in vitro-grown *N. nuda* to SA for 7 days and 28 days, while examining the effects of a broader range of SA concentrations, to optimize conditions for the stimulation of iridoid accumulation in *N*. *nuda*. Future studies should evaluate if SA-induced responses could influence the iridoid and phenolic profile of *N. nuda* under field conditions, as our results indicate optimal concentrations and exposure times of this natural plant hormone for stimulating NL and RA accumulation in in vitro systems. In addition, it would be valuable to examine the effect of lower SA concentrations, as it might further enhance *trans*,*trans*-NL accumulation while minimizing potential stress effects.

The concentration and stereoisomeric composition of NL in *N. nuda* L. varies significantly in response to elicitors and environmental factors. Petrović et al. [21] demonstrate that waterlogging and hydrogen-peroxide elicitation can affect overall iridoid and phenolic content in *N. nuda* by modulating the expression of iridoid and RA biosynthetic genes. Petrova et al. [12] report that in vitro cultivation of *N. nuda* under variable light conditions modulates the levels of several bioactive compounds, including aesculin, ferulic acid, RA, cirsimaritin, naringenin, rutin, isoquercitrin, 1,5,9-*e*DLA, and CGA. Furthermore, the content of NL in *N. nuda* plants can differ between organs and tissues, and in response to various endogenous factors [12,13]. Zhiponova et al. [52] demonstrate that the cytokinin 6-benzylaminopurine (BAP) induces callus formation of *N. nuda* plants grown under in vitro conditions, which is accompanied by increased accumulation of RA and CA, but with no changes in iridoid content. Methyl jasmonate (MeJA) treatments significantly increased the content of major iridoids in *N. rtanjensis* [53]. Dmitrović et al. [54] found that exposure to synthetic compounds such as tetraoxane and thiophene can alter NL levels in *N. cataria* and *N. nuda* without affecting RA content. Considerable metabolic plasticity of *N. nuda* regarding NL and RA production is likely driven by regulatory mechanisms related to iridoid and phenolic acids biosynthesis. Such plasticity makes this species an ideal candidate for elicitation studies, aimed at elucidating the molecular mechanisms underlying upregulation of bioactive compounds’ biosynthesis.

### 2.1. Quantitative UHPLC/DAD/(±)HESI-MS^2^ Analysis of Targeted Iridoids

Besides NL, previous studies have reported various iridoids in *N. nuda* methanol extracts. These include iridoid aglycones such as 5,9-DNL, nepetalactol (NLL), dihydronepetalactone (DHN), and iridoid glycosides—Nnu, aucubin (AU), nepetaside (NS), 1,5,9-*e*DLA, and many others [12,13,21,22,52]. Notably, NL is found in significantly higher concentrations in the leaves of in vitro-grown *N. nuda* plants, compared to wild-grown *N. nuda* [55], which was the rationale for performing experiments under controlled in vitro conditions in this study.

**Figure 2:**
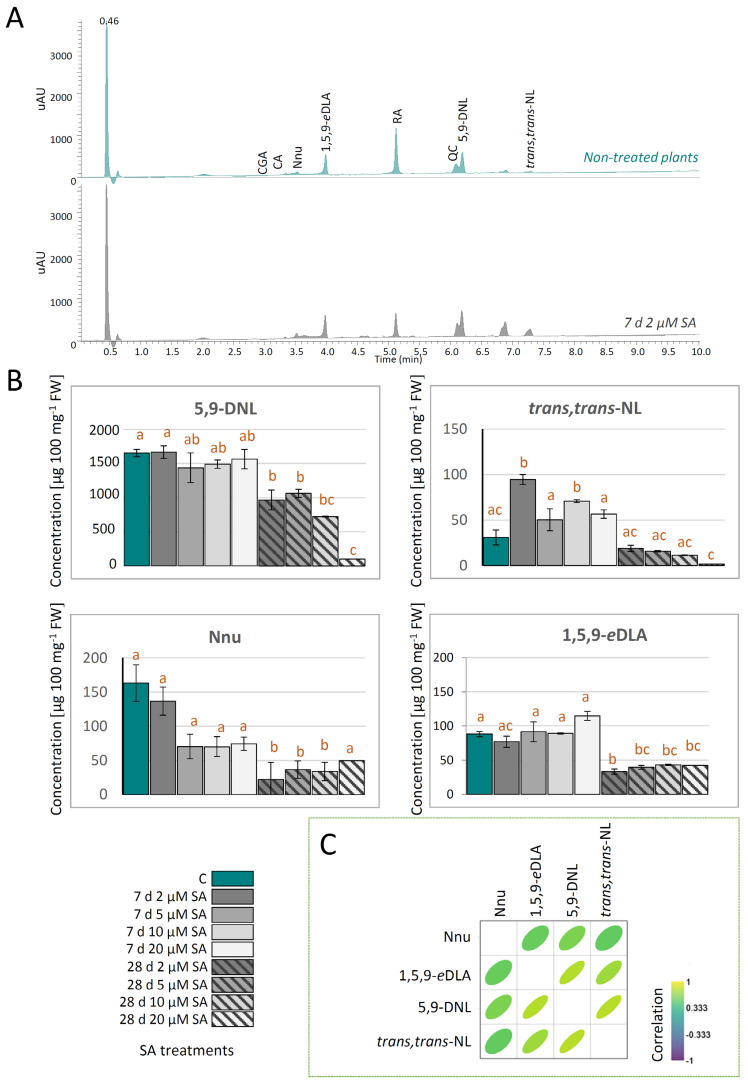
(**A**) Representative UHPLC/DAD chromatograms (λ_max_ = 260 nm) of methanol extracts of non-treated *N. nuda* plants (dark cyan line) and of those exposed to 2 µM salicylic acid (SA) for 7 days (dark gray line). (**B**) Quantitative UHPLC/DAD/(±)HESI-MS^2^ data of targeted iridoid aglycones—5,9-dehydronepetalactone (5,9-DNL) and *trans*,*trans*-nepetalactone (*trans*,*trans*-NL), and of iridoid glycosides—nepetanudoside (Nnu), and 1,5,9-*epi*-deoxyloganic acid (1,5,9-*e*DLA)—in *N. nuda* plants, 7 and 28 days following SA treatments. Bars labeled with different letters are significantly different (*p* < 0.05) according to post hoc Tukey’s test of one-way ANOVA. (**C**) Pearson’s correlations based on the quantitative metabolomics data for 4 targeted metabolites. Positive and negative correlations are presented by the intensity of yellow and blue color, as indicated on the color scale. Correlation plot was constructed using Past5 software, version 4.17. Abbreviations: CA—caffeic acid; CGA—chlorogenic acid; RA—rosmarinic acid; QC—quercetin.

UHPLC/DAD(±)HESI-MS/MS analysis was targeted towards major iridoid aglycones in *N. nuda*, NL and 5,9-DNL, and the iridoid glycosides (Nnu and 1,5,9-*e*DLA). Representative UHPLC/DAD chromatograms at λ_max_ = 260 nm are presented in Figure 2A. SA treatment induced significant changes in the accumulation of iridoid compounds in *N. nuda*, with distinct effects depending on the concentration and duration of exposure (Figure 2B). The content of *trans*,*trans*-NL showed a marked increase following 7 days of exposure to all applied SA concentrations, with the most significant accumulation occurring at 2 µM and 10 µM SA treatments. However, the content of NL was decreased on 28-day treatments, and the most pronounced reduction occurred on treatments with 20 µM SA (Figure 2B). This suggests that the effects of SA on NL biosynthesis and accumulation are both dose- and time-dependent. The concentration of 5,9-DNL remained stable across all SA treatments following 7-day exposure, but showed a significant concentration-dependent decline after 28 days of SA treatments, particularly at the highest applied concentration (20 µM SA). This pattern parallels the decrease in *trans*,*trans*-NL content, indicating that prolonged exposure to 20 µM SA strongly inhibits the metabolic flux through the aglycone branch of the iridoid biosynthetic pathway. The iridoid glycosides showed slightly different responses to SA treatment compared to the iridoid aglycones, suggesting partially independent regulation of the two iridoid pathway branches. It has previously been reported that the most abundant IGs of the 11-COOH type in *N. nuda* are 1,5,9-*e*DLA and Nnu [56]. After 7 days of SA treatments, Nnu levels showed a slight, but statistically non-significant decrease across applied concentrations (Figure 2B). However, this decline became more pronounced after 28 days of exposure to SA. Similarly, concentration of 1,5,9-*e*DLA remained relatively stable to different SA concentrations after 7 days of exposure. On the other hand, a prolonged exposure of *N. nuda* plants to SA resulted in a consistent decline in 1,5,9-*e*DLA content across all treatments. The correlation analysis based on quantitative metabolomics data revealed only positive correlations between the amounts of targeted iridoids following SA elicitation treatments (Figure 2C). The significant positive correlations were recorded between *trans*,*trans*-NL and 5,9-DNL. As suggested, 5,9-DNL arises following spontaneous dehydrogenation of the NL skeleton or by the activity of yet unidentified enzymes [56]. Lockhart et al. [57] consider 5,9-DNL as a degradation product of NL formed during the extraction processes. Understanding the origin of 5,9-DNL is in the course of our future work. Statistically significant positive correlations were also observed between 1,5,9-*e*DLA and 5,9-DNL. Not surprisingly, the amounts of the two IGs targeted in this study—Nnu and 1,5,9-*e*DLA—were also significantly positively correlated. As previously suggested, 1,5,9-*e*DLA is one of the precursors in the biosynthesis of Nnu, which arises after the oxidation of 1,5,9-*e*DLA and subsequent dehydrogenation of *epi*-loganic acid [56].

Based on the quantitative metabolomics data in the present study, which point to the most prominent increase in *trans*,*trans*-NL content in *N. nuda* plants after 7 days of exposure to 2 µM SA, this treatment is highlighted here as an efficient way to elicit NL accumulation. Overall, the obtained results highlight that short-term exposure to SA can have a stimulating effect on NL production, without notably changing the production of other targeted iridoids. In contrast to the study by Allen et al. [39], which reports no increase in NL yield following a 24 h exposure of *N. cataria* to SA, our results suggest that a 7-day exposure period provides sufficient time for activation of iridoid biosynthetic pathway components and for achieving measurable accumulation of NL in plants. However, extension of the SA exposure period to 28 days appears to suppress the accumulation of iridoid compounds, particularly at higher SA concentrations.

### 2.2. Co-Expression Patterns of Nepetalactone Biosynthesis-Related Genes as Influenced by Salicylic Acid Treatments

**Figure 3:**
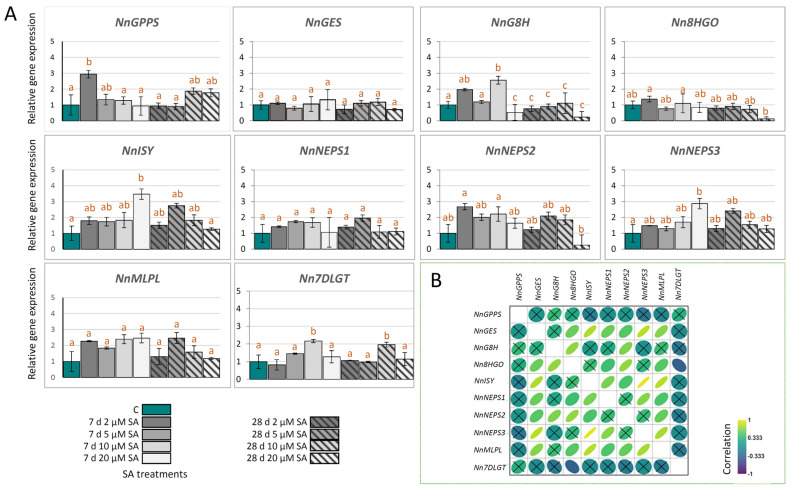
(**A**) The expression of nepetalactone biosynthesis-related genes in leaves of *N. nuda* 7 days and 28 days following salicylic acid (SA) treatments (2, 5, 10, and 20 µM). Obtained values were normalized against *NnGAPDH* as the endogenous control and are presented relative to respective control (expression in non-treated plants represented by dark cyan bars) as calibrators. Different SA treatments are presented by different colors and patterns, as indicated on the symbol legend. Different letters denote significantly different values (*p* < 0.05) according to post hoc Tukey’s test following one-way ANOVA. (**B**) Pearson’s correlations based on the expression levels of 10 iridoid biosynthetic genes. Non-significant correlation values are crossed (*p* > 0.05). Positive and negative correlations are presented by the intensity of yellow and blue color, as indicated on the color scale. Correlation plot was constructed using Past5 software, version 4.17. Abbreviations: GPPS—geranyl diphosphate synthase; GES—geraniol synthase; G8H—geraniol 8-hydroxylase; 8HGO—8-hydroxygeraniol oxidoreductase; ISY—iridoid synthase; NEPS—NAD-dependent nepetalactol-related short-chain dehydrogenase/reductase; MLPL—major latex protein-like enzyme; 7DLGT—7-deoxyloganetic acid glucosyltransferase.

In order to explain the molecular background of the recorded metabolomic changes, we analyzed co-expression patterns of the iridoid biosynthesis-related genes following SA elicitation (Figure 3A). This analysis includes homologues of previously characterized iridoid-related genes.

After 7 days of SA treatment, *NnGPPS* expression was significantly upregulated solely at 2 µM SA, whereas *NnGES* expression remained unaffected across all treatments (Figure 3A). Expression of *NnG8H* was downregulated after 7 days of exposure of *N. nuda* plants to 20 µM SA, while a significant increase in the expression of this gene was recorded at 10 µM SA (Figure 3A). Short-term exposure of *N. nuda* plants to higher SA concentrations also significantly stimulated the expression of genes encoding for *Nn*ISY and *Nn*NEPS3 (20 µM SA). The expressions of *NnNEPS1* and *NnMLPL* were not significantly affected by short-term SA treatments, while *NnNEPS2* was downregulated only at 20 µM SA. Long-term (28 days) exposure of *N. nuda* plants to different SA concentrations in culture media resulted in the downregulation of *NnG8H*, *Nn8HGO*, and *NnNEPS2* in leaves in the presence of 20 µM SA. On the other hand, the expression of other analyzed genes was not affected by long-term SA treatments. In another study, 24 h exposure of *N. cataria* plants to 500 µM and 1000 µM SA resulted in no changes in the NL content, although significant upregulation of *GPPS* and *NEPS1* expression was recorded. Shorter exposures (4 and 12 h) to SA had no effect on gene expression and NL content [39]. In light of the results of the present study, it is obvious that species-specific optimization of the elicitation conditions is necessary.

Previous studies dealing with representatives of the genus *Nepeta* have revealed coordinated expression patterns of iridoid biosynthesis-related genes in response to different abiotic and biotic agents, endogenous factors, and elicitors [21,52,58,59]. Thus, iridoid biosynthetic genes were coordinately up- or downregulated in leaves of *N. rtanjensis* and *N. argolica* following dehydration [58] or in *N. rtanjensis* and *N. nervosa* following MeJA treatment [53]. In *N. sibirica*, a great portion of iridoid-related biosynthetic genes were co-expressed in response to elicitation induced by *Trichoderma harzianum* and *T*. *viride* infection [59]. In the present study, Pearson’s correlation analysis suggests that *NnGES*, *NnISY*, *NnNEPS3* and *NnMLPL* are mostly significantly positively correlated with other iridoid biosynthesis-related genes (Figure 3B). On the other hand, *NnGPPS* and *Nn7DLGT* showed no significant correlations with other genes. The only significantly negative correlation was observed for the expression of *Nn8HGO* and *Nn7DLGT* (Figure 3B).

The gene expression levels were significantly positively correlated with the levels of iridoids in *N. nuda* [13], *N. rtanjensis* and *N. argolica* [58], and in *N. sibirica* [59]. This was also the case for *N. nuda* exposed to waterlogging stress and hydrogen peroxide elicitation [21]. In the present study, linear regression analysis reveals that the levels of some of the major iridoids are correlated with the expression levels of *NnGPPS*, *NnG8H*, *NnISY*, *NnMLPL* and *Nn7DLGT* after 7 days of SA exposure, while no correlation was observed between iridoid biosynthetic genes and iridoid levels following prolonged SA exposure (Table 1). The obtained results are consistent with the above-mentioned studies and align with the observed NL profile, further supporting the relationship between iridoid gene expression and iridoid accumulation.

These results indicate dynamic changes in *N. nuda* metabolism in response to SA exposure. Observed variations in the content of major iridoids, as influenced by the SA concentration and treatment duration, can be explained by perturbed expression of corresponding biosynthetic genes. While lower concentrations of SA, especially following short-term exposure (7 days), tend to stimulate certain iridoid biosynthetic genes (*NnGPPS*, *NnG8H*, *NnISY*, *NnNEPS1*, *NnNEPS2*, *NnNEPS3*, and *NnMLPL*), prolonged exposure at 20 µM SA appears to suppress the expression of genes such as *NnG8H* and *Nn8HGO*. The observed downregulation of *NnG8H* and *Nn8HGO* is, most likely, responsible for the decreased metabolic flux through the iridoid pathway, and thus for the reduced content of iridoids in *N. nuda* leaves, particularly iridoid aglycones. Conversely, the slight upregulation of *NnISY*, *NnNEPSs*, and *NnMLPL*, which are involved in the last few biosynthetic steps preceding the formation of NL, during short-term exposure to SA, points to the significant role of these enzymes in determining NL productivity in *N. nuda* leaves.

### 2.3. Quantitative UHPLC/DAD/(±)HESI-MS^2^ Analysis of Targeted Phenolics

Besides RA, highlighted as a dominant phenolic compound in Nepeta species, the phenolic profile of *N. nuda* includes compounds such as CA, CGA, aesculin, ferulic acid, rutin, quercetin (QC) and luteolin (LU), although in much lower quantities [11,12,13,21,22,60]. However, these compounds surely contribute to the overall bioactive properties of plants enhancing their medicinal potential, particularly in combating oxidative stress and inflammation.

**Figure 4:**
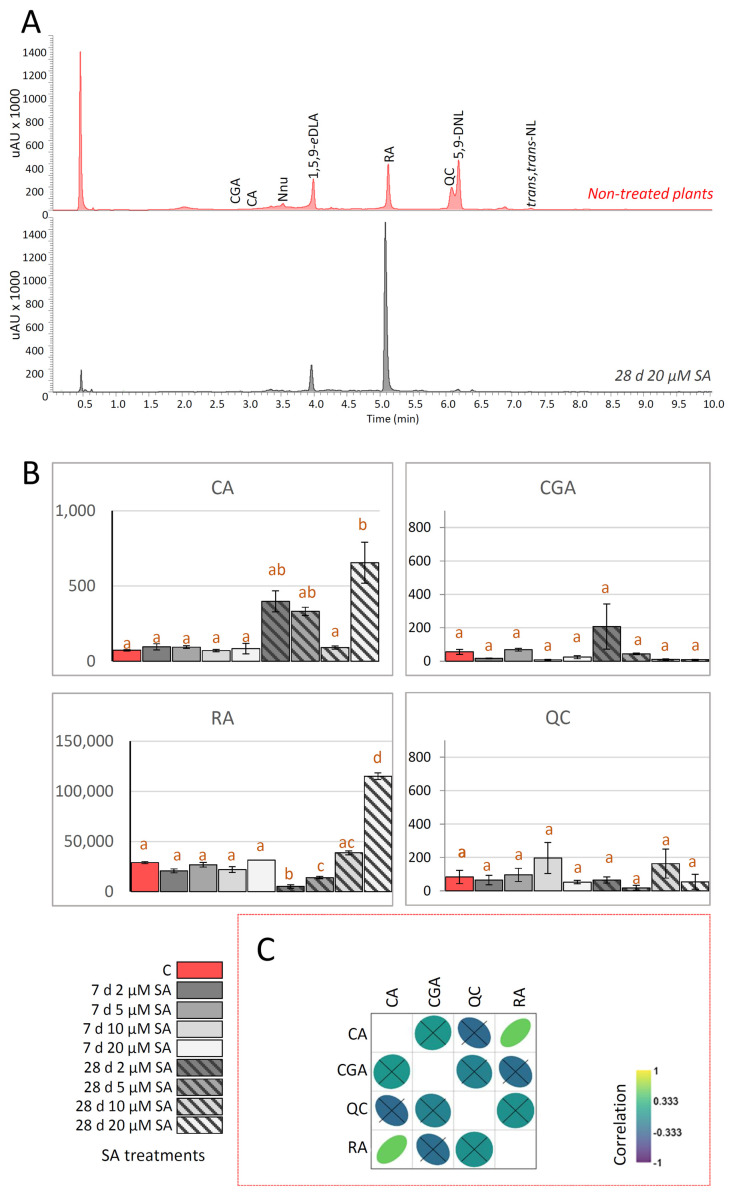
(**A**) Representative UHPLC/DAD chromatograms (λ_max_ = 260 nm) of methanol extracts of non-treated *N. nuda* plants (orange red line) and of those exposed to 20 µM salicylic acid (SA) for 28 days (dark gray line). (**B**) Quantitative UHPLC/DAD/(±)HESI-MS^2^ data of targeted phenolics in *N. nuda* plants, 7 and 28 days following SA treatments. Different SA treatments are presented by different colors and shapes, as indicated on the symbol legend. Bars with different letters are significantly different (*p* < 0.05) according to post hoc Tukey’s test of one-way ANOVA. (**C**) Pearson’s correlations based on the quantitative metabolomics data for 4 targeted metabolites. Positive and negative correlations are presented by the intensity of yellow and blue color, as indicated on the color scale. Non-significant correlation values are crossed (*p* > 0.05). Correlation plot was constructed using Past5 software, version 4.17. Abbreviations: CA—caffeic acid; CGA—chlorogenic acid; RA—rosmarinic acid; QC—quercetin.

Not surprisingly, RA emerged as the most abundant phenolic compound in *N. nuda* leaves analyzed in this study (Figure 4A,B), with significantly lower quantities of CA, CGA, and QC being detected. Short-term exposure to SA did not significantly alter RA, CA, CGA and QC levels in leaves of *N. nuda*. While certain concentrations of SA modulated the content of RA and CA in *N. nuda* following 28 days of the experiment, no statistically significant changes were observed for CGA and QC content. Prolonged exposure led to a notable increase in RA and CA content at 20 µM SA. Interestingly, the prolonged exposure to 2 and 5 µM SA resulted in a significant decrease in RA content in *N. nuda* (Figure 4B). These findings suggest that, contrary to iridoids, extended exposure of *N. nuda* to high SA concentrations promotes an increase in the accumulation of major phenolics, such as RA and CA. Similarly, application of 10 μM SA via culture media for 8 weeks resulted in an increase in RA levels in *Thymus membranaceus*, which further improved the antioxidant properties. Tretament with 100 μM SA led to the decerase of RA content in *T. membranaceus*, while 1000 μM SA decreased the viability of treated explants [61]. Treatment with 50 μM SA for 6 weeks enhanced RA accumulation and antioxidant activity in *Thymus lotocephalus* [62]. However, in the present study the same concetration (50 μM SA) negatively affected *N. nuda* in vitro growth and development (Supplementary Materials, Figure S1), indicating that *Nepeta* species are more sensitive to higher doses of SA. Our findings indicate that prolonged exposure to 20 µM SA is necessary in order to stimulate the phenolic metabolism of *N. nuda*.

The correlation analysis based on the quantitative metabolic data acquired in this study reveals only one statistically significant positive correlation, between RA and CA (Figure 4C). Considering that RA is an ester of CA and 3,4-dihydroxyphenyllactic acid [63], this was not surprising. The majority of observed correlations were not statistically significant, which is not unexpected, given the structural differences between the targeted phenolic compounds, and the fact that they originate from distinct branches of the complex and highly ramified phenolic biosynthetic network in plants. Our findings indicate that individual phenolics may respond differently in SA-induced modulation of phenolic metabolism in *N. nuda*, highlighting the complexity of phenolic biosynthesis.

### 2.4. Expression of Rosmarinic Acid-Related Biosynthetic Genes Under Salicylic Acid Treatment

**Figure 5:**
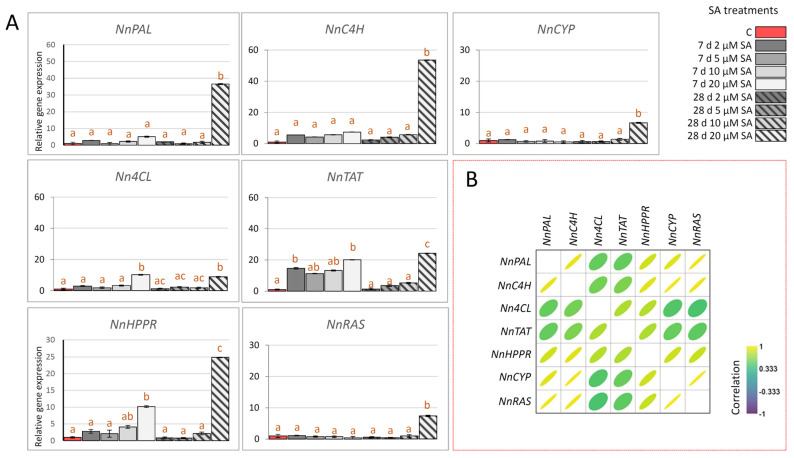
(**A**) The expression of RA biosynthetic genes in leaves of *N. nuda* 7 days and 28 days following SA treatments (2, 5, 10, and 20 µM). Obtained values were normalized against *NnGAPDH* as the endogenous controls and are presented relative to respective control (expression in non-treated plants is represented by dark orange bars) as calibrators. (**B**) Pearson’s correlations based on the expression levels of 7 rosmarinic acid biosynthesis-related genes. Positive and negative correlations are presented by the intensity of yellow and blue color, as indicated on the color scale. Correlation plot was constructed using Past5 software, version 4.17. Bars with different letters are significantly different (*p* < 0.05) according to post hoc Tukey’s test of one-way ANOVA. Abbreviations: PAL—phenylalanine ammonia-lyase; C4H—cinnamic acid 4-hydroxylase; 4CL—4-coumaric acid CoA-ligase; TAT—tyrosine aminotransferase; HPPR—hydroxyphenylpyruvate reductase; RAS—4-coumaroyl-CoA:4′-hydroxyphenyllactic acid 4-coumaroyltransferase; CYP—cytochrome P450-dependent monooxygenase.

We analyzed the expression of homologs of seven key RA biosynthesis-related genes (*NnTAT*, *NnHPPR*, *NnC4H*, *Nn4CL*, *NnPAL*, *NnCYP*, and *NnRAS*) in leaves of *N. nuda* exposed to SA elicitation for 7 and 28 days (Figure 5A). Notably, all genes exhibited their highest levels of upregulation following 28 days of exposure to 20 µM SA, which coincided with the highest levels of RA and CA recorded in *N. nuda* samples. Short-term (7 days) exposure of *N. nuda* to different concentrations of SA showed no stimulatory effect on the expression of *NnPAL*, *NnC4H*, *NnCYP*, and *NnRAS* (Figure 5A). The expression of *Nn4CL* and *NnHPPR* was significantly upregulated after short-term exposure to 20 µM SA. On the other hand, *NnTAT* exhibited upregulation with 2 and 20 µM SA short-term exposure. Interestingly, upregulation of some of the RA biosynthetic genes (*Nn4CL*, *NnTAT*, and *NnHPPR*) after 7 days of exposure to 20 µM SA did not affect the content of RA or other targeted phenolics in *N. nuda* plants. This indicates that *NnRAS* and *NnCYP*, which are directly involved in the final steps of RA biosynthesis, may be critical points for increasing RA levels. These results underscore the importance of *NnRAS* and *NnCYP* as potential candidates for metabolic engineering aimed at enhancing RA production in *Nepeta* species. Furthermore, as *PAL*, *C4H*, and *4CL* are part of the general phenylpropanoid pathway, and *TAT* and *HPPR* regulate tyrosine metabolism and are early-stage enzymes in RA biosynthesis [64], their upregulation may have driven the production of other metabolites, not targeted in this study, without affecting RA levels. Similarly, it has been shown that SA upregulates the expression of *TAT*, *PAL*, and *RAS* genes in *Salvia miltiorrhiza* suspension cells, which results in enhanced accumulation of phenolic acids, including RA and CA [45]. In another study, the expression of *PAL* genes is modulated in *S. officinalis* and *S. virgata* shoots following SA elicitation [65]. SA promotes the interaction between kinase activity of mitogen-activated protein kinase 3 (SmMAPK3) and SmRAS1 in *S. miltiorrhiza* hairy roots, activates the kinase activity of SmMAPK3, and enhances the phosphorylation level and protein stability of SmRAS1, which results in increased contents of RA and its derivative salvianolic acid [66]. In safflower (*Carthamus tinctorius*), an oilseed industrial medicinal plant, SA induces the expression of the *C4H* gene and the accumulation of phenolic compounds in a time- and concentration-dependent manner [67]. *C4H* transcription was also increased in *Ginkgo biloba* following various abiotic stresses and phytohormones, including SA [68].

Pearson correlation analysis shows that expressions of targeted RA biosynthesis-related genes are significantly positively correlated (Figure 5B), revealing their coordinated regulation in response to SA elicitation. It has been previously reported for a number of the Lamiaceae family representatives that RA biosynthesis-related genes are co-expressed in response to various stresses, phytohormones, and elicitors, as well as in different organs, which alters the accumulation of RA and other phenolic compounds [13,52]. Zhiponova et al. [52] showed that the exogenous application of the phytohormone 6-benzylaminopurine (BAP) induces coordinated changes in the expression levels of genes involved in the biosynthesis of RA and CA in in vitro-grown *N. nuda* plants. Furthermore, significant positive correlations were observed between the expression levels of *NnCYP* and *NnRAS* in different *N. nuda* organs, characterized by distinct phenolic profiles [13].

Linear regression analysis reveals no statistically significant correlations between the expression of RA biosynthesis-related genes and the content of targeted phenolics in analyzed *N. nuda* samples following SA treatments (Table 2). The exception is the correlation between *NnHPPR* expression and RA content after prolonged SA exposure, which was statistically significant. This is not surprising, taking into account that the majority of analyzed genes are related to the general phenylpropanoid pathway and are thus involved in providing the precursors for the biosynthesis of various classes of phenolic compounds. Furthermore, such results might be a result of the fact that SA usually induces immediate responses in RA-related biosynthetic gene expressions, while accumulation of metabolites usually takes more time. Similarly, linear regression analysis revealed no significant correlations between the content of RA and the expression of RA-related biosynthetic genes in *N. nuda* shoots exposed to waterlogging stress and H_2_O_2_ elicitation [21]. Petrović et al. [13] reveal that the expression levels of *NnPAL*, *NnC4H*, *Nn4CL*, *NnRAS* and *NnCYP* are not significantly correlated with the RA content in leaves and inflorescences of *N. nuda*. Furthermore, no positive correlations between the intensity of *PAL* transcription and the RA accumulation in *S. officinalis* and *S. virgata* plants exposed to SA elicitation were observed [65]. However, Song et al. [69] show that in *S. miltiorrhiza*, transcript levels of *4CL2* were mainly correlated with RA content under modulated light conditions, while the expression of *PAL1*, *C4H*, and *HPPR* is directly linked to the accumulation of both RA and salvianolic acid B (SAB). On the other hand, *TAT* and *4CL1* were not statistically correlated with either product [69].

Although the present study focuses on the effect of salicylic acid (SA) on iridoid and phenolic production in in vitro-grown *N. nuda* plants, comparable stimulatory effects on specialized metabolite production have been documented in other *Nepeta* and Lamiaceae species under field conditions with foliar application [41,70,71,72] and in hydroponic systems with SA added to the nutrient solution [39]. Obviously, optimization of both the concentration and duration of SA application is essential to achieve maximum effects on phenolic and terpene compound production while avoiding any negative or toxic effects of SA on plant growth and development [73]. However, it is clear that SA can effectively promote terpene and phenolic biosynthesis in Lamiaceae species regardless of the mode of application, whether incorporated into the culture medium or applied foliarly, highlighting its versatile potential for both controlled in vitro production and practical open-field cultivation. Furthermore, considering that SA has the potential to improve plant resistance to challenging environmental conditions, its application may be significant for sustainable agriculture under such conditions [38].

## 3. Materials and Methods

### 3.1. Plant Material and Growth Conditions

Seeds of *N. nuda* were collected in July 2022 from natural populations at the locality Debeli Lug in Serbia (44°21′45″ N; 21°54′01″ E). Plants were identified by the authors, and voucher specimen (No. 17854) was deposited in the Herbarium of the Institute of Botany and Botanical Garden, University of Belgrade, BEOU (acronym follows Thiers, 2016 [74]). The seeds were surface sterilized in a 20% commercial bleach solution for 10 min, then thoroughly rinsed five times with sterile deionized water. Subsequently, they were placed on plastic Petri dishes (9 cm diameter) containing 20 mL of half-strength Murashige and Skoog (MS) culture medium [75], referred to as basal medium (BM). This BM was modified to contain half-strength macroelements, 20 g L^−1^ sucrose, and 7 g L^−1^ agar (Torlak, Belgrade, Serbia). To obtain sufficient plant material for experiments, micropropagation of a selected single *Nepeta nuda* genotype was performed using one-node stem segments as explants. Explants were sub-cultured onto fresh BM every four weeks. All cultures were maintained in the laboratory for plant tissue culture at the Institute for Biological Research “Siniša Stanković” (Belgrade, Serbia) under controlled temperature conditions of 25 ± 2 °C and a 16/8 h light/dark photoperiod, with light provided by white fluorescent tubes displaying photon flux density of 70 µmol m^−2^ s^−1^.

### 3.2. Experimental Setup

The effect of SA on the iridoid and phenolic metabolism of *N. nuda* was evaluated by supplying BM with five different SA concentrations (2 µM, 5 µM, 10 µM, 20 µM, 50 µM). SA working solutions were filter-sterilized through a 0.2 µm cellulose filter (Agilent Technologies, Santa Clara, CA, USA) and added to the BM after sterilization by autoclaving at 114 °C for 25 min and cooling to room temperature. Five nodal segments of *N. nuda* were placed in each 300 mL glass jar containing 50 mL BM, supplemented with SA. The control group of plants was grown on BM without SA supplementation. The experimental conditions were identical to those used for the micropropagation of the selected single *N. nuda* genotype, as described above.

The experiment was conducted in two sample subsets: (1) plants were continuously grown on BM with different SA concentrations for 28 days; (2) plants were initially grown on BM with SA for 7 days, and subsequently transferred to BM without SA. In both cases, plant material was collected on the 28th day, immediately frozen in liquid nitrogen (LN), and stored at −80 °C until further analyses. Since 50 µM SA had a negative effect on plant growth and development, this subset was excluded from further analysis (Supplementary Materials, Figure S1). Results were obtained from three independent biological replicates. Each biological replicate consisted of leaf tissue harvested from five *N. nuda* shoots grown in a single glass jar.

### 3.3. Preparation of N. nuda Methanol Extracts

Freshly frozen *N. nuda* leaves (50 mg) were ground into a fine powder using liquid nitrogen and then dissolved in 1 mL of 96% methanol. The samples were subjected to ultrasonic extraction for 30 min at 4 °C and were centrifuged at 12,000× *g* for 15 min. The resulting supernatants were filtered through 0.2 μm syringe filters (Agilent Technologies, Santa Clara, CA, USA) and stored at 4 °C until further analysis. All procedures were performed in three biological replicates, for each SA treatment.

### 3.4. UHPLC/DAD/(±)HESI-MS^2^ Quantification of Targeted Iridoids and Phenolics in N. nuda Leaves

The quantification of major iridoids—*trans*,*trans*-NL, 5,9-DNL, nepetanudoside (Nnu), 1,5,9-*e*DLA, and phenolics—RA, CA, CGA, and QC—in the leaves of in vitro-grown *N. nuda* (both SA-treated and non-treated plants) was conducted using a Dionex UltiMate 3000 UHPLC system equipped with a diode-array detector (DAD) and coupled to a triple-quadrupole mass spectrometer (TSQ Quantum Access Max, Thermo Fisher Scientific, Braunschweig, Germany). The quantification was performed in selected reaction monitoring (SRM) mode of the UHPLC/DAD/(±)HESI-MS^2^ instrument, in negative (RA, CA, CGA, QC, 1,5,9-*e*DLA, and Nnu) or positive ionization mode (*trans*,*trans*-NL and 5,9-DNL). Chromatographic separations of the samples (10 µL injection volume) were performed on a Hypersil Gold C18 analytical column (50 × 2.1 mm, 1.9 µm particle size; Thermo Fisher Scientific, Germany), maintained at a constant temperature of 40 °C. The mobile phase consisted of water with 0.1% formic acid (solvent A) and acetonitrile with 0.1% formic acid (solvent B), and the flow rate was set to 0.300 mL min^−1^. The elution gradient of the mobile phase and the settings of the mass spectrometer were previously described [13,70]. Chromatographic and MS data for targeted metabolites are presented in Table S1. Quantification of iridoid and phenolic compounds in samples was performed based on the calibration curves of pure standards, as described by [76]. *Trans*,*trans*-NL was quantified based on the calibration curve of *cis*,*trans*-NL, while Nnu was quantified using the calibration curve of loganin (Sigma-Aldrich, Hamburg, Germany). All calibration curves demonstrated excellent linearity, with r^2^ values exceeding 0.99 (peak area vs. concentration). The total amount of each compound was calculated based on its peak area and expressed as µg per 100 mg of fresh leaf weight. All analyses were carried out in three biological replicates.

### 3.5. Statistical Analysis of the Targeted Metabolomics Data

Quantitative metabolomics data, for the comparison between different SA treatments, were subjected to Tukey’s post hoc test (* *p* < 0.05) following one-way ANOVA. Correlation analysis using Ward’s algorithm was conducted in Past 5 software (version 5.2.1, Natural History Museum, University of Oslo) to assess associations between the quantities of the analyzed metabolites in elicitation experiments.

### 3.6. Chemicals and Reagents

Solvents for HPLC/MS analyses, acetonitrile (Fisher Scientific, Leicestershire, UK) and formic acid (Merck, Darmstadt, Germany) were of MS grade. Ultra-pure deionized water was generated using a Water Purification System (New Human Power I Integrate, Human Corporation, Seoul, Republic of Korea). Standards of *cis,trans*-nepetalactone (*cis*,*trans*-NL), *trans,cis*-nepetalactone (*trans*,*cis*-NL), 1,5,9-*epi*-deoxyloganic acid (1,5,9-*e*DLA), and 5,9-dehydronepetalactone (5,9-DNL) were isolated from natural sources as previously described by [70]. Commercially available analytical standards of loganin (LO), rosmarinic acid (RA), caffeic acid (CA), chlorogenic acid (CGA), luteolin (LU) and quercetin (QC) (Sigma-Aldrich, Hamburg, Germany) were used for quantification purposes.

### 3.7. RNA Extraction and Gene Expression Analysis

Total RNA was isolated from *N. nuda* leaves following a modified CTAB protocol described by [77]. RNA quantity was measured using both an N60 Nano-Photometer^®^ (Implen GmbH, Munich, Germany) and a Qubit 3.0 Fluorometer (Thermo Fisher Scientific, Waltham, MA, USA). To eliminate any potential genomic DNA contamination, RNA samples were treated with DNase I (Thermo Fisher Scientific, USA) at 37 °C for 30 min. For reverse transcription, the RevertAid First Strand cDNA Synthesis Kit (Thermo Fisher Scientific, USA) was employed according to the manufacturer’s protocol, using oligo(dT) primers to generate cDNA. Candidate genes related to the nepetalactone and rosmarinic acid biosynthetic pathways in *N. nuda* have been previously reported [21]. Gene expression analysis was performed using a real-time PCR (qPCR) on the QuantStudio™ 3 Real-Time PCR System (Life Technologies, Carlsbad, CA, USA). Thermocycling conditions were optimized as follows: an initial denaturation at 95 °C for 10 min, followed by 40 cycles of 95 °C for 15 s, 60 °C for 30 s, and 72 °C for 30 s, with a final extension at 72 °C for 10 min. Each qPCR reaction contained Maxima SYBR Green/ROX Master Mix (2X) (Thermo Fisher Scientific, USA), with cDNA equivalent to 50 ng of RNA, and 0.3 μM of each primer, according to the manufacturer’s instructions. Expression levels of the putative NL- and RA-related biosynthetic genes were calculated using the 2^−ΔΔCt^ method [78], using GAPDH as the reference gene. All the analyses were performed in three biological replicates, and were normalized to the values recorded for the non-treated group of plants.

### 3.8. Statistical Analysis of Gene Expression Data

For the comparison between treatments, gene expression values were subjected to Tukey’s post hoc test (* *p* < 0.05) following one-way ANOVA. Correlation analysis using Ward’s algorithm was conducted using Past 5 software (version 5.2.1, Natural History Museum, University of Oslo), to assess co-expression patterns of the selected biosynthetic genes in *N. nuda* leaves as affected by SA treatments. Linear regression analysis was applied to examine relationships between metabolomic and gene expression data, and coefficients of determination (R^2^) were calculated using the above-mentioned version of the Past 5 software.

## 4. Conclusions

This study provides valuable insights into the metabolic plasticity of *N. nuda*, demonstrates the effectiveness of SA in optimizing NL and RA production, and explains the molecular background of elicitation effects by pointing to the key regulatory genes determining the metabolic flux through the two biosynthetic pathways. The obtained results suggest that the duration of SA treatment and the applied concentration determine the intricate balance between stimulatory and inhibitory effects of SA on NL and RA metabolism in the leaves of in vitro-grown *N. nuda*. A 7-day SA treatment, particularly at 2 µM, was shown to effectively enhance *trans*,*trans*-NL production, whereas prolonged exposure for 28 days at 20 µM SA emerged as the optimal condition for eliciting RA biosynthesis. The differential responses of the two biosynthetic pathways to SA elicitation underscore the complexity of their regulatory mechanisms and highlight the necessity for independent fine-tuning of elicitation conditions to achieve optimal yields of these two groups of bioactive compounds. By identifying potential gene targets for metabolic engineering—namely *NnNEPS2* involved in NL biosynthesis and the RA-related genes *NnCYP* and *NnRAS*—this study offers practical implications for improving bioactive compounds production in *N. nuda*. Overall, providing alternative sources of *Nepeta* iridoids and phenolics may enhance their future application in biological control and the pharmaceutical industry.

## Figures and Tables

**Table 1:** Correlations (R^2^) between quantitative metabolomics data for selected iridoid metabolites and qPCR expression data for 10 iridoid biosynthetic genes after 7 days (black) and 28 days (red) of salicylic acid treatment. Asterisks denote significantly different values in respect to the non-treated plants, according to the *t*-test (* *p* < 0.05).

Genes	Targeted Iridoids			
	5,9-DNL	*trans*,*trans*-NL	Nnu	1,5,9-*e*DLA
*NnGPPS*	0.000229	0.33046 *	0.074293	0.17256
	0.2248	0.21294	0.009546	0.0059124
*NnGES*	0.006704	0.0083651	0.066854	0.064565
	0.14549	0.14029	0.020209	0.064735
*NnG8H*	0.10939 *	0.154	0.017494	0.39118
	0.17693	0.12214	0.043614	0.010777
*Nn8HGO*	0.010985	0.074856	0.004397	0.15411
	0.6327	0.47338	0.065118	0.079632
*NnISY*	0.16303	0.00002	0.33252 *	0.05708
	0.00255	0.00086231	0.15539	0.11563
*NnNEPS1*	0.14734	0.0034214	0.17934	0.00033772
	0.003765	0.0014777	0.13339	0.041011
*NnNEPS2*	0.07789	0.096699	0.10813	0.12734
	0.12652	0.09169	0.011032	0.0054566
*NnNEPS3*	0.042552	0.0030417	0.18568	0.14522
	0.00004	0.0020319	0.13987	0.060176
*NnMLPL*	0.020259	0.1733 *	0.24445	0.023744
	0.028963	0.014095	0.074359	0.018242
*Nn7DLGT*	0.00006	0.038507 *	0.007597	0.00072587
	0.38718	0.40841	0.11864	0.067379

Abbreviations: GPPS—geranyl diphosphate synthase; GES—geraniol synthase; G8H—geraniol 8-hydroxylase; 8HGO—8-hydroxygeraniol oxidoreductase; ISY—iridoid synthase; NEPS—NAD-dependent nepetalactol-related short-chain dehydrogenase/reductase; MLPL—major latex protein-like enzyme; 7DLGT—7-deoxyloganetic acid glucosyltransferase; 5,9-DNL—5,9-dehydronepetalactone; *trans*,*trans*-NL—*trans*,*trans*-nepetalactone; Nnu—nepetanudoside; 1,5,9-*e*DLA—1,5,9-*epi*-deoxyloganic acid.

**Table 2:** Correlations (R^2^) between quantitative metabolomics data for selected phenolics and qPCR expression data for 7 RA biosynthetic genes after 7 days (black) and 28 days (red) of salicylic acid treatment. Asterisks denote significantly different values in respect to the non-treated plants, according to the *t*-test (* *p* < 0.05).

Genes	Targeted Phenolics			
	CA	CGA	QC	RA
*NnPAL*	0.15483	0.094052	0.010944	0.057373
	0.36894	0.040612	0.0004325	0.80403
*NnC4H*	0.00003	0.25361	0.0003106	0.000272
	0.5477	0.054434	0.0051277	0.8752
*Nn4CL*	0.051687	0.043597	0.039046	0.1278
	0.58551	0.069726	0.03594	0.77585
*NnTAT*	0.004188	0.15692	0.015678	0.000705
	0.53605	0.091837	0.026305	0.85607
*NnHPPR*	0.08477	0.026644	0.0069326	0.12691
	0.51083	0.051055	0.0042564	0.91009 *
*NnRAS*	0.04547	0.036822	0.11871	0.066191
	0.453	0.080384	0.0072202	0.91494
*NnCYP*	0.003008	0.10902	0.078202	0.097227
	0.3707	0.048747	0.0015765	0.89127

Abbreviations: PAL—phenylalanine ammonia-lyase; C4H—cinnamic acid 4-hydroxylase; 4CL—4-coumaric acid CoA-ligase; TAT—tyrosine aminotransferase; HPPR—hydroxyphenylpyruvate reductase; RAS—4-coumaroyl-CoA:4′-hydroxyphenyllactic acid 4-coumaroyltransferase; CYP—cytochrome P450-dependent monooxygenase; CA—caffeic acid; CGA—chlorogenic acid; QC—quercetin; RA—rosmarinic acid.

## Data Availability

The original contributions presented in this study are included in the article/Supplementary Materials. Further inquiries can be directed to the corresponding authors.

## Supplementary Materials

The following supporting information can be downloaded at: https://www.mdpi.com/article/10.3390/ijms27083570/s1.
