# Supplementary material for: Salicylic Acid-Induced Elicitation of Nepetalactone and Rosmarinic Acid Biosynthesis in Naked Catmint (Nepeta nuda L.): Metabolomic and Transcriptional Insights
Source: Int J Mol Sci. 2026 Apr 16;27(8):3570. doi: 10.3390/ijms27083570 (PMC13115748; doi:10.3390/ijms27083570)
Supplement: Supplementary file 1 [file ijms-27-03570-s001.zip › ijms-4201189-supplementary/Figure S1.pdf]

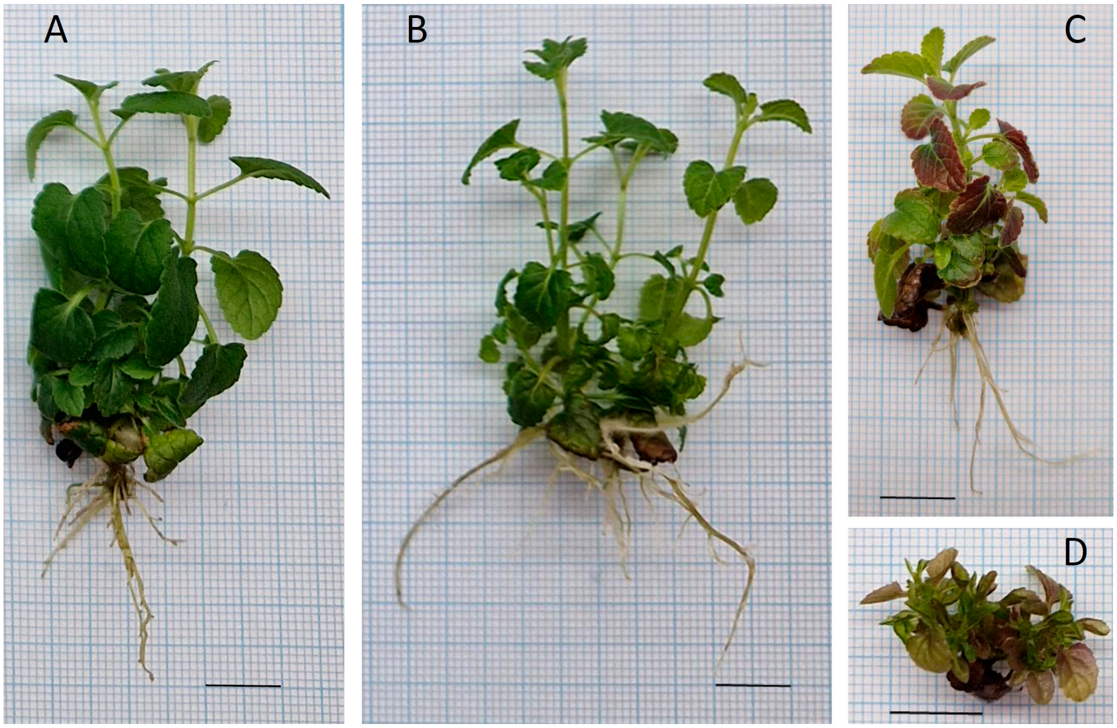


**Supplementary Figure S1.** Plants of *Nepeta nuda* cultured *in vitro*. Presented are: (A) plant grown on basal medium (BM, half-strength Murashige and Skoog medium) - control; (B) plant grown on BM supplemented with 2 μM salicylic acid (SA) for 7 days and subsequently transferred to BM without SA for three weeks; (C) plant continuously grown for 28 days on BM with 20 μM SA; (D) plant continuously grown for 28 days on BM with 50 μM SA. Plants were placed on a graph paper, where a black line represents 1 cm.
