# Supplementary material for: Salicylic Acid-Induced Elicitation of Nepetalactone and Rosmarinic Acid Biosynthesis in Naked Catmint (Nepeta nuda L.): Metabolomic and Transcriptional Insights
Source: Int J Mol Sci. 2026 Apr 16;27(8):3570. doi: 10.3390/ijms27083570 (PMC13115748; doi:10.3390/ijms27083570)
Supplement: Supplementary file 1 [file ijms-27-03570-s001.zip › ijms-4201189-supplementary/Table S1.pdf]

Table S1. Chromatographic and mass spectrometry data of the adopted UHPLC/DAD/(±)HESI–MS^2 analytical method for the quantification of eight targeted phenolics and iridoids in methanol extracts of *N. nuda* plants.

		**UHPLC/MS data**				**UHPLC/DAD data**	
	Metabolite	t_R [min]	$[M–H]^–$ [m/z]	Diagnostic MS^2 fragments [m/z]	cE [eV]	t_R [min]	λ_{max} [nm]
**1**	Chlorogenic acid[a]	2.76	353	191, 127	30	2.64	230, 330
**2**	Caffeic acid[a]	3.02	179	135, 134	30	2.90	230, 330
**3**	Loganin[a]	3.43	435*	227, 127	30	3.31	230
**4**	Nepetanudoside[R]	3.59	435*	227, 101	30	3.47	230, 330
**5**	1,5,9-*epi*-Deoxyloganic acid[a]	4.09	405*	359, 197	30	3.97	240
**6**	Rosmarinic acid[a]	5.38	359	161, 133	30	5.26	240, 330
**7**	Quercetin[a]	6.40	301	179, 151	30	6.28	270, 360
	Metabolite	t_R [min]	$[M+H]^+$ [m/z]	Diagnostic MS^2 fragments [m/z]	cE [eV]	t_R [min]	λ_{max} [nm]
**8**	5,9-Dehydronepetalactone[a]	6.31	165	149, 77	20	6.19	300
**9**	*trans*,*trans*-Nepetalactone[R]	7.23	167	111, 77	20	7.11	225
**10**	*cis*,*trans*-Nepetalactone[a]	7.34	167	111, 77	20	7.22	225

Abbreviations: *- Visible as an adduct with formic acid $[M+HCOOH–H]^–$, cE- collision energy, [a]- Identified using authentic standards, [R]- Confirmed by references; t_R- retention time.
